# How to influence the continuous usage intention of game-based Internet public welfare users? An empirical analysis based on SEM and fsQCA

**DOI:** 10.1371/journal.pone.0325933

**Published:** 2025-06-11

**Authors:** Jing Liu, Xiaohan Chen, Xuwei Zhou, Jianxiang Wei, Chengzhi Liu

**Affiliations:** 1 School of Management, Nanjing University of Posts and Telecommunications, Nanjing, China; 2 School of Computer Science, The University of Sydney, Sydney, Australia; 3 Library, Nanjing University of Posts and Telecommunications, Nanjing, China; Tsinghua University, CHINA

## Abstract

This study focuses on UTAUT and integrates the D&M, SDT, and SET models to explore the factors influencing continuous usage intention in the context of gamified online public welfare. By applying structural equation modeling and fuzzy-set qualitative comparative analysis to 389 valid survey samples, this study identifies that public value, leisure and entertainment, and self-payment significantly influence users’ continuous usage intention. Furthermore, game design and tool convenience, along with subjective norms, are key drivers of these three factors. In contrast, gaming convenience and social factors have limited impact on user engagement. The findings reveal three enhancing configurations—functionality-driven, comprehensive support, and pure public value paths—and four inhibiting configurations—complete disengagement, social impact without incentives, convenience without enjoyment, and engagement without enjoyment paths. The crucial role of subjective norms is found to be consistently significant in both the SEM and fsQCA results, making it a key factor in promoting user engagement in gamified public welfare activities. This research contributes to the theoretical understanding of user participation in game-based charity activities and provides insights for improving user retention. Its originality lies in the integration of SDT and SET models alongside D&M and UTAUT, as well as the use of mixed methodologies to examine the multifaceted drivers of continuous usage intention.

## 1 Introduction

In the digital age, the Internet offers opportunities for public interest organizations to connect with their target audiences. Game-based Internet public welfare campaigns are characterized by their widespread appeal, ease of access and high transparency [1], as they enhance the achievement of public welfare projects’ goals and values by integrating game elements into non-game contexts, thus stimulating users’ participation and sharing behaviors. The 2022 Internet Good Summit emphasized the ‘establishment of a national unified charitable big data platform to facilitate the sharing of charitable donation and relief data’ as a significant mission for the public welfare industry and technology enterprises during their dialogue on digitalization of public welfare.

At present, an increasing number of individuals are participating in gamified online public welfare campaigns. Statistics demonstrate that since ‘Ant Forest’ went online in 2016, it has enabled over 650 million people to adopt low-carbon lifestyles, planted more than 400 million trees across 4.5 million acres, and supported public welfare organizations in developing 24 eco-conservation projects to protect over 1,600 wildlife species. Since its launch in 2007, Freerice has contributed over 200 million kilograms of rice to famine-stricken regions worldwide, providing the equivalent of 120 million meals. EVOKE, initiated in 2010, has drawn nearly 20,000 players from over 120 countries, and proposed over 14,000 innovative solutions addressing global challenges such as poverty, hunger, health and education. These statistics underline that game-based online public welfare has become a highly popular mode of contributing to the public good.

Existing research on gamified online public welfare campaigns primarily focuses on describing their characteristics rather than analyzing users’ continuance intention. Furthermore, these studies rely heavily on structural equation modeling (SEM), a method capable of evaluating causality amongst multiple variables, yet lacking in-depth analysis of interaction among various influential factors. Therefore, this study employs both SEM and fuzzy-set qualitative comparative analysis (fsQCA) to investigate continuous usage intention in the context of gamified online public welfare. A theoretical model is established taking into consideration the collective effects of multiple factors. Specifically, this study selects the D&M model (information systems success model) and UTAUT (the unified theory of acceptance and use of technology), well-suited for the Internet context, in order to construct a more appropriate theoretical framework. This research concentrates on continuous participation behaviors in gamified online public welfare campaigns, investigating the motivations and determining factors that drive continuous user engagement. The outcomes are expected to provide valuable insights into factors influencing user engagement with gamified online public welfare, thereby promoting practical advancements within this field.

This study aims to address the following research questions related to game-based Internet public welfare campaigns:

Existing research on gamified online public welfare campaigns tends to focus on describing their characteristics rather than analyzing users’ continuance intention. Furthermore, these studies rely entirely on structural equation modeling (SEM), a method capable of evaluating causality amongst multiple variables, yet lacking in-depth analysis of interaction among various influential factors. Therefore, this study employs both SEM and fuzzy-set qualitative comparative analysis (fsQCA) to investigate continuous usage intention aligned with gamified online public welfare. A theoretical model is established taking into consideration the collective effects of multiple factors. Specifically, this study selects the D&M model (Delone&Mclean information systems success model), UTAUT (the unified theory of acceptance and use of technology), SDT (self-determination theory), and SET (social exchange theory), apt for the Internet context, in order to construct a more comprehensive theoretical framework. This research concentrates on continuous participation behaviors in gamified online public welfare campaigns, investigating the motivations and determining factors that drive continuous user engagement. The outcomes are expected to offer significant insights into factors influencing user engagement with gamified online public welfare, thereby promoting practical advancements within this field.

This study aims to address the following research questions related to game-based Internet public welfare campaigns

- What factors can affect continuous usage intention?
- How does the interaction of these factors collectively impact the formation of continuous usage intention?
- What are the main factors that can affect continuous usage intention?

## 2 Literature review

### 2.1 Internet community research

The characteristics of community and gamification, standing out as distinctive features of game-based Internet public welfare, significantly influence user engagement and retention [2]. Community traits refer to the diversity of users in terms of backgrounds, interests, motivations, and behaviors, leading to a wide range of user experiences and engagement levels. For instance, a community with a shared interest or goal may engender a sense of belonging among its members, thereby enhancing continuous user engagement. Gamification involves the application of game-design elements in non-game scenarios. Within the framework of Internet public welfare, gamification can render the experience more entertaining, engaging, and rewarding for users. Nonetheless, due to the diversity among communities and the extensive range of available content, the intention to consistently utilize game-based Internet public welfare platforms and the factors influencing this intention can vary greatly across users [3]. This underscores the need for a nuanced understanding of user behavior and the development of personalized approaches to enhance user engagement and retention. Several researchers have advanced theories that may provide such an understanding, shedding light how users form their identities within these communities (communication theory of identity) [4], exhibit immersion and engagement during participation (flow theory) [5], and how their perceptions and emotions influence their level of involvement (cognitive theory of emotion) [6]. Furthermore, these theories highlight the purposeful and systematic nature of user behavior (theory of planned behavior) [7], along with the motivation of users’ needs and satisfactions in opting for specific media and activities (uses and gratifications approach) [8,9], and elucidate how users acquire and adapt within social settings is elucidated (social cognitive theory) [10]. Additionally, the value of social networks (social capital theory) [11] and the importance of trust and commitment for user retention (commitment-trust theory) [12] are also revealed. These theoretical frameworks provide a profound foundation for understanding the complexity and diversity of user behavior on gamified public welfare platforms.

The prolonged utilization of various online communities and platforms is indeed a multifaceted phenomenon that has been explored through different sociological and psychological perspectives. Moreover, the motivations driving users to continuously engage with these platforms are diverse and often context-specific. For instance, in health communities, users’ primary motivation is their need for health-related information [13]. In brand communities, the emphasis is on fostering social interactions between consumers and brands, which nurtures a sense of belonging and loyalty [14]. In the case of the online video platform BILIBILI, user retention is enhanced by actively engaging users in the governance of the platform, thereby creating a sense of ownership and commitment [15]. For digital archive communities, members’ continuous use is motivated by a public desire to participate in the social development of archival resources, contributing to a shared cultural heritage [16]. Lastly, the activity within Q&A communities is largely dependent on the need for knowledge sharing, where users find value in both asking questions and providing answers [17]. These examples illustrate the wide range of factors that can influence the continuous use of online communities, highlighting the importance of understanding the specific context and user needs of each platform.

Existing research on internet communities often focuses on specific aspects of user engagement, such as social identity or motivations for participation, without considering the combined influence of community diversity and gamification elements. Many studies explore how shared interests or goals enhance user engagement in niche communities, but these studies typically do not account for how different user profiles interact with gamified features within broader online public welfare platforms. Unlike previous works, which tend to isolate community characteristics or game mechanics, this study examines how both dimensions work together to influence sustained user engagement in game-based internet public welfare. By combining community diversity with gamification, this research offers a more holistic understanding of user behavior and retention, addressing the gap in literature regarding the complex interplay of these factors.

### 2.2 Gamified online charity campaign characteristics research

Gamified online public welfare campaigns indeed provide a unique approach to engaging individuals in social responsibility activities. By integrating game elements within the online platform, these campaigns can stimulate individual emotions, create a sense of recognition and belonging, and encourage active participation in public welfare [18]. The core of this model lies in the effective use of gamification’s denotative properties, which are the explicit, observable elements of a game, such as points, badges, leaderboards, and challenges [19]. The inclusion of game features has the potential to enhance users’ enjoyment and engagement, thus increasing their motivation to participate [20].

Moreover, by offering an entertaining and interactive platform, these gamified campaigns can significantly lower the barrier to public participation. They create a simpler and more enjoyable way for individuals to contribute to public welfare, even if they have no prior experience or knowledge in this area [21], having the potential to attract a wider audience and generating more significant impact. Indeed, gamified public welfare campaigns can stimulate individuals to engage not only actively [22] but also persistently [23] in prosocial behaviors, i.e., actions intended to help others or benefit society as a whole. These activities could encompass a variety of actions such as donating, volunteering, or participating in a community cleanup. The mechanism through which public good gamification promotes such behaviors can be partially explained by the mediating role of group norms [24]: shared expectations about how members of a group should behave. Within the frame of gamified public welfare campaigns, these norms may involve expectations about participation levels, the types of activities users engage in, or the ways in which they interact with each other. Group norms that are in line with prosocial behaviors have the potential to significantly impact individual actions. For instance, if it becomes a norm within the community to regularly participate in certain activities or to strive for certain achievements (e.g., earning badges or points), individuals may be more likely to engage in these behaviors on a consistent basis. This can lead to a continuous commitment to prosocial actions, thereby amplifying the impact of the gamified public welfare campaign. During gameplay, the enjoyment and competitive elements of gameplay are crucial factors influencing players’ adoption of online gaming for public welfare aims [25]. The interplay between enjoyment, competitive preferences, and the promotion of eco-friendly behaviors in proenvironmental games is not static, but varies depending on the state of the game [26]. In parallel, the rise of the Internet has led to a significant transformation in the public’s engagement with charity and public welfare. Traditional habits have been reshaped, with online platforms now playing a dominant role in the allocation and management of social resources [27]. The Internet, with its extensive reach and large user bases, effectively overcomes geographical limitations [28]. It also addresses issues of information asymmetry and resource scarcity, paving the way for more efficient and equitable distribution of resources [29]. This digital shift fosters a positive interaction dynamic between those offering help (suppliers) and those in need of it (demanders). Moreover, the Internet revolutionizes the channels through which people participate in public welfare [30], providing a platform where users can express their values and identify with environmental and public welfare causes. This is achieved through a blend of online and offline prosocial engagement, enabling users to communicate their commitment to these issues more effectively [31]. In this context, public welfare games can be seen as a unique intersection of these trends, utilizing the principles of enjoyment and competition to promote public welfare behaviors. This highlights the potential of such platforms in fostering a culture of environmental consciousness and active participation in public welfare.

In summary, while significant strides have been made in understanding the factors influencing continuous usage intention in online communities, there remain considerable gaps in the current body of research. Existing studies have predominantly focused on the impact of community characteristics, regularly neglecting diversity in usage intentions and the interplay between multiple influencing factors. Moreover, although previous research has explored continuous usage intention across various contexts, employing a range of sociological and psychological perspectives, there has been a lack of focus on how the unique contexts provided by the Internet itself shape user behaviors and attitudes. This has resulted in an incomplete understanding of the dynamics of user engagement in online settings. Additionally, the unique characteristics of the Internet landscape and its users have not been adequately considered. The idiosyncrasies of online settings, which can significantly influence user engagement and interaction, have often been overlooked, leaving a significant gap in our knowledge. This highlights the need for future research to consider the specificities of online environments when examining continuous engagement in internet communities and gamified platforms. By doing so, we can gain a more nuanced understanding of user behaviors and attitudes in these settings, which can inform the design and implementation of more effective engagement strategies.

### 2.3 D&M, UTAUT, SDT and SET

The DeLone and McLean information systems success model (D&M model) is used to evaluate the success of information systems across a range of metrics. In 2003, DeLone and McLean proposed an improved version of the original model, consisting of six variables: information quality, system quality, service quality, use, user satisfaction, and net benefits [32]. This improved model has since been widely applied in information system evaluations. For example, service quality, interface quality, and content quality were found to significantly influence user satisfaction in mobile reading applications [33]. Additionally, information quality positively affects community group buying usefulness perceptions and satisfaction [34]. Moreover, shopping website service quality, recommender system quality, and recommendation information quality were found to impact recommendation adoption intention through the mediators perceived usefulness and ease of use [35]. Such applications across Internet communities indicate the D&M model’s suitability for this context.

The unified theory of acceptance and use of technology (UTAUT) was proposed by Venkatesh et al. in 2003 [36], positing four variables impacting usage intention: performance expectancy, effort expectancy, social influence, and facilitating conditions. UTAUT has since been widely leveraged to examine internet use intentions across contexts such as reading [37], health [38], financial management [39], office settings [40], and travel [41]. For instance, effort expectancy, performance expectancy, task/technology match, social influence, and facilitating conditions play vital roles in mobile banking user adoption [42].

Self-Determination Theory (SDT), developed by Deci and Ryan in the 1980s, focuses on intrinsic and extrinsic motivation and the conditions that support or undermine human autonomy, competence, and relatedness [43]. According to SDT, users are motivated by their need for autonomy, competence, and relatedness, and these needs can significantly influence their behavior in digital environments [44]. In the context of gamified platforms, SDT suggests that users’ intrinsic motivation (e.g., enjoyment and personal growth) and extrinsic motivation (e.g., rewards and recognition) play crucial roles in sustaining engagement. Research has applied SDT to examine user motivations in video game [45], social media [46], and educational apps [47], highlighting the importance of satisfying these basic psychological needs to foster long-term participation.

Social Exchange Theory (SET) posits that human relationships are based on a series of exchanges that involve the assessment of benefits and costs [48]. In the context of technology acceptance,users engage with platforms when they perceive the benefits (e.g., entertainment, social recognition, or functional utility) to outweigh the costs (e.g., time investment or privacy concerns) [49]. SET has been applied to understand user behavior in online communities [50], social networks [51], and online purchase [52]. In gamified public welfare platforms, SET implies that users are more likely to continue their participation if they perceive that the social and functional benefits provided by the platform outweigh any perceived costs.

Although existing research has provided valuable insights into the drivers of user engagement, several limitations are evident. Firstly, many studies tend to apply theories such as D&M, UTAUT, SDT, and SET in isolation, often focusing on individual aspects like user satisfaction, technology adoption, or behavioral intention, without considering how these models interact to capture the complex and multifaceted nature of sustained user participation. This fragmented approach overlooks the dynamic interplay between factors such as functionality, social influence, and intrinsic motivation, which are crucial for understanding long-term engagement in gamified public welfare platforms. Secondly, while these studies often address specific contexts, such as commercial technology adoption, they fail to generalize findings to the online public welfare sector, where motivations and behaviors can differ significantly. Additionally, many studies neglect the critical relationship between platform design—such as system quality and usability—and the psychological factors influencing engagement. While D&M and UTAUT emphasize system quality and convenience, they often overlook the role of intrinsic motivations and social responsibility, key factors in the public welfare context. Moreover, existing models are typically applied to well-established commercial settings, leaving a gap in their applicability to non-profit or socially-driven platforms. Building upon the Unified Theory of Acceptance and Use of Technology(UTAUT) as the foundational framework, this study establishes an integrated analytical model centered on motivation mechanisms. The selection of UTAUT as the core stems from its proven efficacy in explaining technology adoption behaviors, particularly its emphasis on performance expectancy and social influence—two critical dimensions in public welfare gamification contexts. We extend this foundation through strategic incorporation of complementary theories: D&M Model operationalizes UTAUT’s facilitating conditions by quantifying system quality and information quality, creating measurable indicators for technical infrastructure assessment. Self-Determination Theory(SDT) enriches the effort expectancy construct by distinguishing between intrinsic motivation (leisure enjoyment) and identified regulation, explaining how gamification elements satisfy psychological needs for autonomy and competence. Social Exchange Theory(SET) recontextualizes social influence as reciprocal relationship building, where users’ self-payment is balanced against perceived social capital gains, aligning with UTAUT’s social influence dimension through the lens of mutual obligation.

This integration transcends mere theoretical juxtaposition by establishing causal linkages: Technical infrastructure(D&M) enables motivation formation(SDT), which is moderated by social dynamics(SET). The framework thereby addresses the core challenge of sustaining engagement in prosocial technologies, where utilitarian acceptance(UTAUT) must evolve into value-driven commitment(SDT/SET).

### 2.4 Research hypotheses

**Fig 1:**
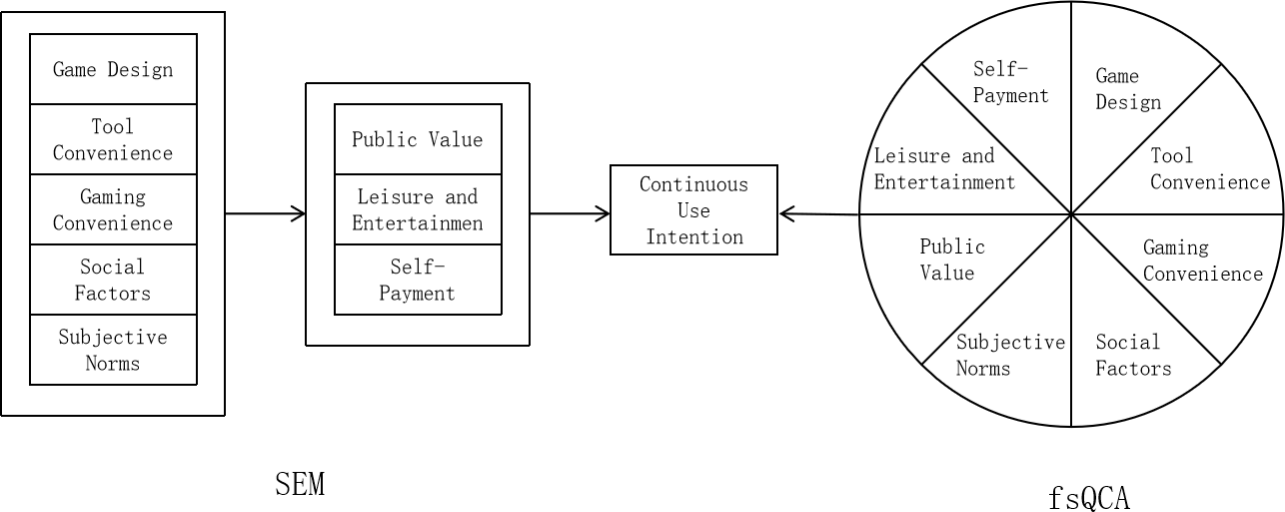
Research model.

Drawing from the information system success dimensions within the D&M model, the four drivers of usage motivation in the UTAUT model, and the intrinsic and extrinsic motivations highlighted in the SDT and SET theories, this paper proposes a research model of continuous usage intention (UI) in the context of gamified online public welfare campaigns. The model includes eight factors: game design (GD), tool convenience (TC), gaming convenience (GC), social factors (SF), subjective norms (SN), public value (PV), leisure and entertainment (LE), and self-payment (SP), as shown in Fig 1.

According to the research model, the following hypotheses are

H1: Game design has a significant positive impact on public value.

H2: Game design has a significant positive impact on leisure and entertainment.

H3: Game design has a significant positive impact on self-payment.

H4: Tool convenience has a significant positive impact on public value.

H5: Tool convenience has a significant positive impact on leisure and entertainment.

H6: Tool convenience has a significant positive impact on self-payment.

H7: Gaming convenience has a significant positive impact on public value.

H8: Gaming convenience has a significant positive impact on leisure and entertainment.

H9: Gaming convenience has a significant positive impact on self-payment.

H10: Social factors have a significant positive impact on public value.

H11: Social factors have a significant positive impact on leisure and entertainment.

H12: Social factors have a significant positive impact on self-payment.

H13: Subjective norms have a significant positive impact on public value.

H14: Subjective norms have a significant positive impact on leisure and entertainment.

H15: Subjective norms have a significant positive impact on self-payment.

H16: Public value has a significant positive impact on continuous usage intention.

H17: Leisure and entertainment have a significant positive impact on continuous usage intention.

H18: Self-payment has a significant positive impact on continuous usage intention.

## 3 Methodology

### 3.1 Participants

This study conducted a questionnaire survey targeting users of gamified internet philanthropy platforms. The administration of the survey was approved by the Academic Ethics Committee of Nanjing University of Posts and Telecommunications. Before beginning the survey proper, participants were informed in writing that their participation was voluntary, that the information collected would be used for research purposes only, and that all data would be anonymized. They provided their consent via a written opt-in form at the start of the questionnaire. For participants under the age of 18, considering negligible risk to participants, along with the non-sensitive and anonymous nature of the data, as a reason not to specifically seek parental consent.The survey was distributed on WeChat, the largest social networking software in China; QQ, the most popular messaging software in China; and Xiaohongshu, another prominent social media platform. The convenience sampling method targeted classmates, friends, and online acquaintances. Eligible participants (1) had taken part in game-based Internet public welfare and (2) volunteered for the study. To ensure adequate age coverage, the age of respondents was segmented as follows: under 18, 18–25, 26–30, 31–40, 41–50, 51–60, and over 60 years old. While utilizing the maximum likelihood method to estimate the structural equation model, it is recommended that the sample size be approximately five to ten times the quantity of questionnaire items, with a minimal sample size of 200 [53]. A total of 500 questionnaires were collected; invalid questionnaires, including those from ineligible individuals and those with identical responses throughout, were manually screened out. 389 valid questionnaires were retained as the sample for this study.

This study surveyed users of gamified internet philanthropy platforms through WeChat, QQ, and Xiaohongshu, which are popular among younger individuals, particularly those aged 18–30. While this age distribution reflects both the platforms’ user base and the general appeal of public welfare games to younger audiences, it limits the generalizability of the findings. The underrepresentation of older age groups, particularly those over 40, may affect the external validity of the results.

### 3.2 Instrument

This research was conducted empirically, utilizing a questionnaire that incorporated well-established scales derived from previous studies. The scales were modified to suit the specific context of gamified online public welfare campaigns. The initial section of the questionnaire outlines the objective and significance of the research, while the subsequent section focuses on demographic factors such as gender, educational attainment, and professional occupation. In the third section of the questionnaire, a total of 9 latent variables were measured. The questionnaire utilizes a 5-point Likert scale, with 1 representing ‘strongly disagree’, 3 ‘neutral’, and 5 ‘strongly agree’. This study adopted established items from prior studies to ensure the items were both representative and comprehensible, adapting the language to more accurately reflect the characteristics of game-based Internet public welfare campaigns. References and measurement items are detailed in Table 1.

**Table 1:** Factors influencing continuous usage intention for game-based Internet public welfare.

Variant	Question item	Source
Game Design (GD)	Public welfare game function design and page layout is reasonable and beautiful	[54,58,59]
	Public welfare games are simple and intuitive to operate	
	Public welfare games can always show the whole process of public welfare	
Tool Convenience (TC)	I have smart devices for charity games	[54,63,64]
	I can play charity games anytime and anywhere	
	The smart device I use is compatible with charity games	
Gaming Convenience (GC)	I prefer to choose well-known public service games	[54,66]
	I can find help if I have a problem	
	Charity games are rich and diverse in content	
Social Factors (SF)	I use charity games through my friends	[55,68,69]
	I use charity games to promote them through media advertising	
	I use charity games because they come with major software	
Subjective Norms (SN)	I think the charity games are very interesting	[56,71]
	I have a sense of responsibility for the public good	
	Charity games can give me a sense of accomplishment	
Public Value (PV)	I can contribute to the public good	[55,74]
	I can increase my public welfare contribution (environmental protection certificate, donation value, etc.) via charity games	
	Charity games can stimulate my enthusiasm for public welfare	
Leisure and Entertainment (LE)	I can relax and unwind through charity games	[57,76]
	I can interact with my friends through charity games	
	I can enrich my daily life through public welfare games	
Self-Payment (SP)	I can quickly adapt to charity games	[56,80]
	I can use my fragmented time to participate in charity games	
	I can explore other useful activities in real life through charity games	
Continuous Usage Intention (UI)	In the future, I plan to continue to use charity games	[56]
	In the future, I will maintain or even increase the frequency with which I use charity games	
	I would like to recommend charity games to the people around me	

### 3.3 Data collection

The collection of data was conducted via online survey website Questionnaire Star (www.wjx.cn), which operates independently from any institutional system involved in the acquisition of research samples. To ensure the preservation of respondent confidentiality and anonymity, the questionnaire abstained from gathering personal identifiers such as names, email addresses, or phone numbers. The School of Management at Nanjing University of Posts and Telecommunications granted an official license for the project prior to data collection, allowing for the distribution of extensive online questionnaires. Participants were informed about the survey’s purpose and provided with a clear explanation of the game-based online charity activities before they proceeded to complete the questionnaire. This step was taken to ensure that respondents fully comprehended the questionnaire and to guarantee the accuracy of the data collected, thus enabling an authentic representation of user behavior and motivations in these activities. The questionnaires were gathered between November 22, 2024 and December 12, 2024, followed by the expeditious completion of essential screening procedures. Participants were duly informed that they could opt out of the questionnaire at any time. Additionally, participants were provided with the researcher’s contact details for any further inquiries or clarifications.

### 3.4 Data analysis

To examine the hypotheses, this study utilizes partial least squares structural equation modeling with the assistance of SmartPLS 4.0 software.

To determine the synergistic effect that environmental uncertainty factors have on shaping this association, fuzzy-set qualitative comparative analysis (fsQCA) is employed. To accomplish the analysis, this study employs fsQCA 3.0 software, a dedicated tool designed for this type of research.

### 3.5 Study variables

#### 3.5.1 Game design.

Game design is derived from the system quality variable in the D&M model and refers to the integrated design of game-based Internet public good functions [58] and interfaces [59]. The public good function enables the game to display the entire process by which user actions translate to real-life public goods at all times [60], while the interface determines the clarity of the information conveyed and influences user experience [61]. By providing background, progress updates, and goals through game information and feedback, developers help users gain deeper knowledge and concern for the public welfare cause [62]. In the context of SDT, game design also impacts users’ intrinsic motivation, enhancing enjoyment and engagement.

#### 3.5.2 Tool convenience.

Tool convenience is derived from the facilitating conditions dimension in UTAUT and refers to the ease with which users can participate in gamified online public welfare campaigns without being constrained by time, location, or device compatibility. Smart devices enable temporal and spatial flexibility, thus facilitating participation [63]. Moreover, game availability across multiple devices enhances convenience, impacting users’ willingness to engage [64]. This aligns with UTAUT’s effort expectancy dimension, emphasizing the ease of use of the platform.

#### 3.5.3 Gaming convenience.

Gaming convenience refers to the overall ease of user experience while engaging with gamified online public welfare [65], reflecting system usability, accessibility, and compatibility [66]. It aligns with the facilitating conditions dimension in UTAUT, ensuring that users can easily access and navigate the platform [67]. In SDT terms, gaming convenience enhances extrinsic motivation, as users are more likely to continue participating in platforms that provide seamless, enjoyable experiences with minimal barriers to entry.

#### 3.5.4 Social factors.

Social factors represent the social influence dimension in UTAUT. This refers to the social environment and relationships impacting user participation in gamified online public welfare campaigns [68]. Word-of-mouth communication and social media influence can spark users’ interest in trying these platforms [69]. Additionally, media promotions and integrations as features within larger software ecosystems help increase the platform’s visibility and access opportunities, thus reinforcing social influence [70]. According to SET, social factors contribute to the perceived value exchange between the user and the platform.

#### 3.5.5 Subjective norms.

Subjective norms reflect the social influence that affects users’ engagement with gamified online public welfare platforms, as explained in UTAUT [71]. These norms relate to the perceptions of others’ expectations about participation in such activities [72]. Moreover, subjective norms tie into SDT’s external motivations, where social responsibility and the desire to contribute to public welfare influence continued participation. Users are likely to engage more when they feel the social pressure or social validation for doing so, reinforcing both intrinsic and extrinsic motivations [73].

#### 3.5.6 Public value.

Public value reflects user satisfaction with the prosocial elements within gamified online public welfare campaigns. It is drawn from the user satisfaction dimension in the D&M model and is closely related to the perceived social benefits users derive from their participation [74]. In SET, public value acts as a reward or benefit for users, motivating them to engage in the exchange process [75]. Moreover, in SDT, the desire to contribute to social good is a key intrinsic motivator that drives continuous use and commitment to these platforms.

#### 3.5.7 Leisure and entertainment.

Leisure and entertainment refer to the enjoyment and satisfaction users gain from the gaming aspects within gamified online public welfare campaigns. This concept is derived from the user satisfaction dimension in the D&M model and reflects the entertainment value users derive from participation [76]. As part of SDT, leisure and entertainment are crucial extrinsic motivators, as they fulfill users’ need for fun and relaxation [77]. Engaging with the platform provides an enjoyable escape while also offering social interaction and skill development, further reinforcing intrinsic motivation [78].

#### 3.5.8 Self-payment.

Self-payment refers to the personal resources (e.g., time, money, and effort) that users invest when participating in gamified online public welfare campaigns [79]. This draws from UTAUT’s effort expectancy dimension, where users invest their effort to engage with the platform and experience enjoyment [80]. In SET, self-payment can be seen as part of the reciprocal exchange process, where users contribute resources in return for the social value they gain from their participation [81]. This dimension can influence continued engagement if users perceive a balanced return on their investment.

#### 3.5.9 Continuous usage intention.

Continuous usage intention reflects users’ attitudes and behaviors toward future participation after engaging with game-based Internet public welfare campaigns. This variable captures users’ commitment to using the platform over the long term, indicating their positive experience and ongoing willingness to engage. The intention to recommend the platform to others also signals the user’s perceived value and satisfaction, which is crucial for ensuring platform sustainability.

## 4 Results

### 4.1 Sample characteristic analysis

Users of gamified online public welfare campaigns were the research subjects. In total, 500 questionnaires were collected; eliminating invalid entries (completion time < 60 seconds, uniform answers, conspicuous contradictions) left a final sample of 389 responses. Key demographic information is summarized in Table 2.

**Table 2:** Basic demographics of the study sample.

Category	Option	Frequency	Proportion (%)
Gender	Male	152	39.07%
	Female	237	60.93%
Age	Under 18	25	6.43%
	18 ~ 25	156	40.10%
	26 ~ 30	112	28.79%
	31 ~ 40	77	19.79%
	41 ~ 50	11	2.83%
	51 ~ 60	6	1.54%
	Over 60	2	0.51%
Occupation	Full-time student	198	50.90%
	Office worker	102	26.22%
	Professional	57	14.65%
	Homemaker	30	7.71%
	Retiree	2	0.51%
Use history	Under 1 year	132	33.93%
	1–2 years	116	29.82%
	More than 3 years	141	36.25%

### 4.2 Reliability and validity analysis

The reliability of the scale is determined by the internal consistency coefficient (Cronbach’s α), average variance extracted (AVE) and composite reliability (CR). As demonstrated in Table 3, analyses affirm that all variables possess Cronbach’s alpha values surpassing 0.7, (range: 0.730–0.867). The composite reliability, with values between 0.847 and 0.919, exceeds the benchmark of 0.5 [82], indicating high scale reliability and good internal consistency for this study.

**Table 3:** Reliability and convergent validity analysis.

Variable	Measurement item	Factor loading	Cronbach’sα	CR	AVE
GD	GD1	0.847	0.782	0.872	0.694
	GD2	0.869			
	GD3	0.781			
TC	TC1	0.877	0.798	0.882	0.714
	TC2	0.786			
	TC3	0.869			
GC	GC1	0.748	0.731	0.848	0.651
	GC2	0.817			
	GC3	0.851			
SF	SF1	0.777	0.730	0.847	0.649
	SF2	0.826			
	SF3	0.813			
SN	SN1	0.871	0.836	0.901	0.753
	SN2	0.837			
	SN3	0.895			
PV	PV1	0.873	0.816	0.891	0.732
	PV2	0.874			
	PV3	0.819			
LE	LE1	0.884	0.836	0.901	0.753
	LE2	0.869			
	LE3	0.781			
SP	SP1	0.876	0.809	0.887	0.724
	SP2	0.839			
	SP3	0.835			
UI	UI1	0.881	0.867	0.919	0.790
	UI2	0.903			
	UI3	0.882			

As shown in Table 3, all measurement items have standardized factor loadings exceeding 0.5, and average variance extracted (AVE) values for each latent variable surpass 0.5 [82], indicating good convergent validity of the scales in this study. Moreover, as shown in Table 4, according to the Fornell-Larcker criterion, the square root of the AVEs exceeds the correlation coefficients between the variables, demonstrating good discriminant validity. Thus, the measurement instruments demonstrate high reliability and validity, warranting further structural model analysis [83].

**Table 4:** Discriminant validity analysis.

Var.	GD	TC	GC	SF	SN	PV	LE	SP	UI
GD	0.833								
TC	0.656	0.845							
GC	0.732	0.672	0.807						
SF	0.514	0.500	0.507	0.806					
SN	0.767	0.701	0.734	0.548	0.852				
PV	0.698	0.674	0.674	0.457	0.764	0.856			
LE	0.662	0.614	0.690	0.486	0.642	0.681	0.868		
SP	0.753	0.752	0.682	0.514	0.761	0.726	0.635	0.851	
UI	0.713	0.723	0.767	0.475	0.771	0.706	0.679	0.650	0.889

### 4.3 Hypothesis testing

**Fig 2:**
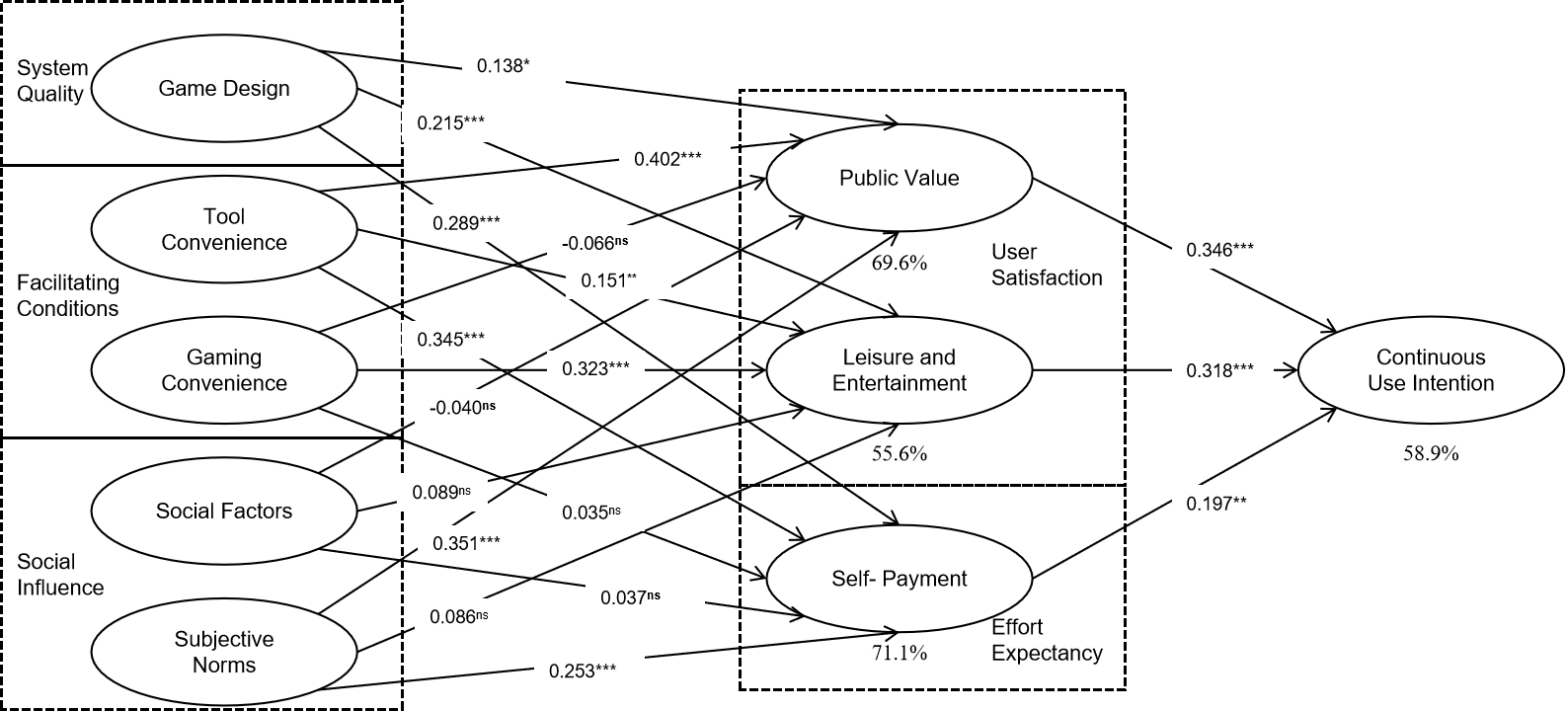
Continuous usage intention model with weights and significance.

The hypothesis testing results are displayed in Table 5. Path analysis reveals that the majority of hypotheses are supported with statistical significance (β > 0, *p *< 0.05) [84], except for H7 (β = 0.066, *p* = 0.166), H9 (β = 0.035, *p* = 0.492), H10 (β = −0.040, *p* = 0.249), H11 (β = 0.089, *p* = 0.059), H12 (β = 0.037, *p* = 0.241)and H14 (β = 0.086, *p* = 0.231) which lack statistical support. Fig 2 provides a detailed visualization of the path analysis results from our hypothesis testing. The figure also highlights hypotheses that did not achieve statistical significance (H7, H9, H10, H11,H12 and H14). These results provide a clear view of the magnitude and direction of influences between variables, helping to understand which hypotheses were supported by the data and which were not.

**Table 5:** Hypothesis Testing.

Hypothesis	Path	β	T	*p*	Conclusion
H1	GD → PV	0.138	2.304	0.021	Supported
H2	GD → LE	0.215	3.548	0.000	Supported
H3	GD → SP	0.289	5.052	0.000	Supported
H4	TC → PV	0.402	8.240	0.000	Supported
H5	TC → LE	0.151	2.958	0.003	Supported
H6	TC → SP	0.345	5.908	0.000	Supported
H7	GC → PV	0.066	1.384	0.166	Not Supported
H8	GC → LE	0.323	4.638	0.000	Supported
H9	GC → SP	0.035	0.686	0.492	Not Supported
H10	SF → PV	−0.040	1.15	0.249	Not Supported
H11	SF → LE	0.089	1.890	0.059	Not Supported
H12	SF → SP	0.037	1.173	0.241	Not Supported
H13	SN → PV	0.351	6.438	0.000	Supported
H14	SN → LE	0.086	1.197	0.231	Not Supported
H15	SN → SP	0.253	4.722	0.000	Supported
H16	PV → UI	0.346	7.069	0.000	Supported
H17	LE → UI	0.318	6.603	0.000	Supported
H18	SP → UI	0.197	3.772	0.002	Supported

### 4.4 Fuzzy-set qualitative comparative analysis

Numerous factors influence participation willingness towards gamified online public welfare campaigns, with complex interrelationships characterized by ‘multiple concurrent causation’ that structural equation modeling alone cannot sufficiently explain through singular causal links. To deeply explore this multifaceted mechanism, this study adopts fuzzy-set qualitative comparative analysis (fsQCA) to examine the antecedents from a configurational perspective, supplementing the net effects analysis from SEM. This explores the mechanisms influencing continuous usage intention within the gamified online public welfare context.

#### 4.4.1 Calibration.

The 5-point Likert scale was converted into a 0-to-1 scale with three calibration anchors set at 5, 3, and 1. Specifically, a value of 5 corresponds to full membership at 0.95; 3 represents the crossover point at 0.5; and a value of 1 denotes full non-membership at 0.05. Table 6 displays excerpted calibration data for selected variables.

**Table 6:** Data Calibration Results (partial).

GD	TC	GC	SF	SN	PV	LE	SP	UI
0.73	0.95	0.35	0.36	0.88	0.95	0.27	0.88	0.73
0.50	0.37	0.50	0.70	0.50	0.50	0.73	0.50	0.50
0.95	0.50	0.95	0.97	0.95	0.88	0.50	0.95	0.50
0.10	0.04	0.35	0.23	0.14	0.10	0.18	0.14	0.14
0.88	0.50	0.73	0.84	0.73	0.50	0.50	0.50	0.88
0.95	0.95	0.95	0.01	0.95	0.95	0.95	0.95	0.95
0.73	0.95	0.35	0.36	0.88	0.95	0.27	0.88	0.73
0.95	0.95	0.95	0.36	0.95	0.95	0.95	0.95	0.95
0.95	0.95	0.95	0.23	0.73	0.95	0.95	0.95	0.95
0.50	0.37	0.23	0.36	0.14	0.18	0.18	0.35	0.35
0.18	0.17	0.23	0.36	0.35	0.10	0.38	0.23	0.23
0.73	0.50	0.50	0.84	0.73	0.88	0.73	0.73	0.35
0.95	0.5	0.95	0.08	0.95	0.95	0.95	0.95	0.95
0.50	0.82	0.73	0.92	0.73	0.73	0.73	0.88	0.88
0.10	0.25	0.35	0.50	0.23	0.18	0.27	0.35	0.23

#### 4.4.2 Analysis of necessary conditions.

The necessity analysis results are displayed in Table 7. All antecedent variables have necessity consistency scores below 0.9 [85], indicating that no individual necessary condition exists in influencing continuous usage intention. Additionally, the necessity analysis for the negation of continuous usage intention also shows that no single necessary condition exists for its non-occurrence. This suggests that the absence of any individual factor does not by itself determine the failure to achieve continuous usage intention.

**Table 7:** Results of necessary condition analysis.

Outcome variable: Continuous usage intention		
Condition Variable	Consistency	Coverage
Game Design	0.826	0.843
~Game Design	0.460	0.558
Tool Convenience	0.777	0.852
~Tool Convenience	0.511	0.572
Gaming Convenience	0.821	0.868
~Gaming Convenience	0.489	0.571
Social Factors	0.771	0.799
~Social Factors	0.512	0.611
Subjective Norms	0.875	0.855
~Subjective Norms	0.427	0.548
Public Value	0.827	0.817
~Public Value	0.442	0.559
Leisure and Entertainment	0.811	0.831
~Leisure and Entertainment	0.484	0.585
Self-Payment	0.840	0.808
~Self-Payment	0.449	0.588
Outcome variable: ~ Continuous usage intention		
Condition Variable	Consistency	Coverage
Game Design	0.547	0.449
~Game Design	0.809	0.789
Tool Convenience	0.526	0.464
~Tool Convenience	0.833	0.750
Gaming Convenience	0.542	0.460
~Gaming Convenience	0.845	0.791
Social Factors	0.594	0.494
~Social Factors	0.759	0.727
Subjective Norms	0.561	0.440
~Subjective Norms	0.815	0.840
Public Value	0.565	0.449
~Public Value	0.769	0.781
Leisure and Entertainment	0.573	0.471
~Leisure and Entertainment	0.794	0.772
Self-Payment	0.608	0.470
~Self-Payment	0.751	0.790

#### 4.4.3 Analysis of sufficient conditions.

The researchers performed sufficiency analysis on the data, parsing the truth table to derive 28 causal combinations. A consistency threshold of 0.8, frequency threshold of 1 case [86], and proportional reduction in inconsistency (PRI) threshold of 0.9 were set [87]. This resulted in 4 configurations meeting the thresholds, as shown in Table 8. All 4 solutions have raw consistencies exceeding 0.9, with an overall solution consistency of 0.948 above the minimum of 0.8, indicating acceptable levels of consistency. Thus, these configurations represent pathways leading to continuous usage intention for gamified online public welfare, accounting for 94.8% of cases. Moreover, with a coverage of 0.624 surpassing 0.5, the explanatory power of these 4 solutions is substantial, explaining 62.4% of outcome membership.

**Table 8:** Results of sufficient condition analysis.

	Continuous Usage Intention			
	Configuration 1	Configuration 2	Configuration 3	Configuration 4
Game Design	●	●	●	●
Tool Convenience	●	●		●
Gaming Convenience	●	●	●	
Social Factors	●		●	
Subjective Norms	●	●	●	●
Public Value		●	●	●
Leisure and Entertainment		●	●	
Self-Payment	●	●	●	●
Original coverage	0.329	0.534	0.507	0.228
Unique coverage	0.030	0.058	0.037	0.018
Consistency	0.958	0.964	0.957	0.970
Total coverage	0.624			
Total consistency	0.948			

Note: (1) “●” and “●” denote the presence of a causal condition, “⊗” its absence, and a blank space indicates the condition is irrelevant (can be either present or absent); (2) “●” signifies a core condition and “●” a peripheral condition.

Configuration 1 suggests that game design, tool convenience, gaming convenience, subjective norms, and self-payment are core conditions, while social factors are considered a peripheral condition. Leisure and Entertainment are not present in this configuration. This configuration explains approximately 33% of continuous usage intention, with about 3% of the intention being uniquely explained by this configuration.

Configuration 2 identifies gaming convenience, subjective norms, and self-payment as core conditions, with game design, tool convenience, public value, and leisure and entertainment as peripheral conditions. It accounts for approximately 53% of continuous usage intention, with about 6% of the intention being uniquely explained by this configuration.

Configuration 3 also has gaming convenience, subjective norms, and self-payment as core conditions, while game design, social factors, public value, and leisure and entertainment are peripheral conditions. This configuration explains around 51% of continuous usage intention, with about 4% of the intention being uniquely explained by this configuration.

Configuration 4 has game design, tool convenience, subjective norms, public value, and self-payment as core conditions, while gaming convenience, social factors, and leisure and entertainment are not present as conditions. Among all the solutions, Configuration 2 demonstrates the highest raw consistency, indicating it is the most effective in activating continuous usage intention for online charity participants. This configuration explains approximately 23% of continuous usage intention, with about 2% uniquely explained by this configuration.

In addition, the analysis was also extended to the negation of continuous usage intention. Similar thresholds and consistency criteria were applied to derive configurations leading to the absence of continuous usage intention. The results indicated that 4 configurations met the thresholds for the negation of continuous usage intention. The overall solution consistency for the negation of continuous usage intention was 0.9487, surpassing the required minimum of 0.8. With a coverage of 0.6062, these configurations explained 60.62% of the cases where continuous usage intention was not achieved (Table 9).

**Table 9:** Results of sufficient condition analysis (the negation of Continuous Usage Intention).

	~Continuous Usage Intention			
	Configuration 1	Configuration 2	Configuration 3	Configuration 4
Game Design	⊗	⊗	⊗	●
Tool Convenience	⊗	⊗	●	⊗
Gaming Convenience	⊗	⊗	⊗	⊗
Social Factors	⊗	●	⊗	●
Subjective Norms	⊗	⊗	⊗	⊗
Public Value	⊗	⊗	●	⊗
Leisure and Entertainment	⊗		⊗	⊗
Self-Payment		⊗	⊗	●
Original coverage	0.521	0.307	0.254	0.255
Unique coverage	0.227	0.021	0.035	0.019
Consistency	0.971	0.952	0.955	0.944
Total coverage	0.606			
Total consistency	0.948			

Configuration 1 for the negation of continuous usage intention includes the presence of none of the following conditions: game design, tool convenience, gaming convenience, social factors, subjective norms, public value, and leisure and entertainment. This configuration explains approximately 52% of the non-occurrence of continuous usage intention, with about 23% uniquely explained by this configuration.

Configuration 2 involves the presence of social factors, while the following conditions do not exist: game design, tool convenience, gaming convenience, subjective norms, public value, and self-payment. This configuration accounts for approximately 31% of the negation of continuous usage intention, with about 2% uniquely explained by this configuration.

Configuration 3 includes the presence of social factors and public value, while game design, tool convenience, and gaming convenience do not exist, along with the absence of leisure and entertainment and self-payment. This configuration explains around 25% of the non-occurrence of continuous usage intention, with approximately 3% uniquely explained by this configuration.

Configuration 4 features the presence of game design and social factors, while tool convenience and gaming convenience do not exist. Additionally, subjective norms, public value, and leisure and entertainment are absent, but self-payment is present. This configuration accounts for about 25% of the negation of continuous usage intention, with approximately 2% uniquely explained.

#### 4.4.4 Robustness analysis.

Robustness checks were conducted by adjusting consistency thresholds (increasing from 0.8 to 0.85) and calibration anchors (90% instead of 95%; 10% instead of 5%). Truth table reconstruction and standardization analysis revealed configuration compositions and research findings to be largely consistent, with only minor metric discrepancies. This evidence demonstrates the reliability and robustness of the analysis results.

## 5 Discussion

This study aims to deepen our understanding of the factors driving continuous usage intention on gamified public welfare platforms by integrating multiple theoretical models, including D&M, UTAUT, SDT, and SET. The findings reveal complex relationships between key variables such as game design, tool and gaming convenience, social factors, subjective norms, public value, self-payment, and leisure and entertainment. While some results confirm expectations, others provide more nuanced insights into user engagement.

### 5.1 SEM results discussion

The relationships between game design and both public value and leisure and entertainment are consistent with existing research [88], reinforcing the importance of high-quality game design in driving user engagement and shaping perceptions of value. Specifically, the positive influence of game design on public value suggests that well-crafted game elements can enhance users’ sense of social responsibility and the platform’s societal impact [89]. This aligns with prior studies that argue game design influences not only entertainment but also the perceived social worth of a platform [90]. The significant impact of game design on leisure and entertainment supports the notion that users are motivated by both enjoyment and intrinsic rewards, in line with SDT, which posits that activities that provide intrinsic satisfaction foster sustained engagement [43].Similarly, the positive influence of tool convenience on public value, leisure and entertainment, and self-payment is consistent with the UTAUT, which highlight the role of ease-of-use and functionality in enhancing user satisfaction and engagement [36]. The convenience of using a platform is a key determinant in fostering a seamless experience, particularly in the context of platforms that require financial contributions, where ease of interaction can directly impact users’ willingness to engage and donate. The relationship between subjective norms and self-payment is also in line with expectations. This suggests that social pressure or perceived social expectations can encourage individuals to contribute financially to the platform, particularly in settings where social validation plays a crucial role in user participation [91]. This reinforces the notion that social influence is a powerful motivator in behaviors related to public goods and charity-based activities. Lastly, the robust positive relationships between public value and continuous usage intention, as well as leisure and entertainment and continuous usage intention, reflect the key role of users’ intrinsic and extrinsic motivations in their long-term engagement with the platform. Public value, by highlighting the societal benefits of participation, aligns with the community-driven aspects of gaming platforms, supporting the idea that users are more likely to continue using platforms that align with their values [92]. The significant relationship between leisure and entertainment and continued usage intention emphasizes that while users are motivated by social causes, the enjoyment factor is equally crucial in driving sustained engagement.

However, several hypotheses did not receive significant support, warranting a more nuanced exploration of the reasons behind these findings. One notable discrepancy is the non-significant relationship between game convenience and public value. Although one might expect that ease of access and gameplay convenience would positively influence users’ perceptions of the public value of a platform, this result suggests a potential misalignment between what users perceive as valuable and the platform’s ease of use. In the context of public welfare gaming platforms, users may prioritize the altruistic value or the mission of the platform over the ease of gameplay. This is particularly evident in the context of online charity-based games, where users’ motivations tend to be more socially driven [93] than functionally driven [92]. Additionally, the minimal influence of game convenience on self-payment could reflect a disconnection between the usability of the game and users’ willingness to contribute. It is possible that users do not see convenience in gameplay as directly correlated with the moral or social satisfaction derived from donating or participating in a cause [94]. Similarly, the non-significant relationship between social factors and both public value and leisure and entertainment may reflect a broader cultural or contextual issue. In many regions, particularly in collectivist cultures, social factors such as peer influence can be crucial for engagement in public welfare activities [95]. However, in this study, the lack of significant effects suggests that the social influence within this specific sample might have been limited or overshadowed by stronger internal motivations such as personal values or enjoyment derived from the platform itself. The absence of these effects may also be attributed to the nature of the platforms surveyed, such as WeChat and QQ, where social interactions are not necessarily central to the user experience. These platforms often serve as utility-based tools, where the primary interaction is with content rather than with other users [96]. Moreover, the weak effect of subjective norms on leisure and entertainment suggests that social expectations do not significantly shape users’ motivation for engagement in a gaming context. While social pressure may encourage individuals to donate or contribute, the drive for entertainment appears to be more autonomous and influenced by intrinsic enjoyment rather than external expectations. This contrasts with research suggesting that peer influence can play a more significant role in leisure-based activities, particularly in online gaming [97]. These results underline the complex interplay between intrinsic and extrinsic motivations in user behavior. While elements like game design, tool convenience, and public value all contributed to sustained usage intentions, other factors such as social influence and game convenience did not exert the expected impact. This raises questions about the changing nature of user motivations in the digital age. In particular, the weak impact of social factors and game convenience suggests that users may be increasingly focused on intrinsic rewards—such as personal satisfaction and social responsibility—over external influences or ease of use. This aligns with research on SDT [98], which posits that intrinsic motivation plays a central role in sustained user engagement, particularly in the context of online platforms with social and charitable objectives.

### 5.2 fsQCA configurations and analysis

The SEM results provide important insights into the direct relationships between key factors influencing continuous usage intention. However, SEM’s linear approach is limited in capturing the complex, multifaceted interactions between conditions. To address this limitation, fsQCA was employed to explore how different combinations of factors lead to sustained user engagement.

#### 5.2.1 Configurations leading to continuous usage intention.

Several configurations of conditions emerged as pathways to sustained engagement:

 Configuration 1 represents a path driven primarily by functionality and social responsibility. This path reflects the importance of clear societal impact and functional design in driving user behavior. In the real world, this approach appeals to users who are more focused on making a positive social contribution than seeking entertainment. Such users are motivated by the societal value the platform provides rather than the entertainment it offers. Platforms using this approach may not need extensive entertainment features but must effectively communicate their societal mission and offer simple yet impactful functional experiences to engage users. This path can be referred to as the “functionality-driven path”, as it emphasizes functionality and social responsibility as the main factors driving sustained engagement, rather than entertainment.

 Configurations 2 and 3 are driven by a comprehensive set of supporting conditions, where the core conditions—gaming convenience, subjective norms, and self-payment—work together to foster sustained usage intention. this path highlights how platforms that balance gaming convenience, subjective norms, and self-payment are better positioned to engage users over the long term. These core conditions align with users’ desire for easy access, social validation, and personal investment, making it more likely that users will return to the platform and continue using it. The supporting conditions, such as tool convenience and public value, play a reinforcing role in maintaining engagement, but they cannot replace the foundational elements that keep users actively participating. Platforms need to ensure that these core conditions are robust and integrated, while also recognizing that entertainment or social interactions, although valuable, do not dominate the overall experience. Thus, this path is best understood as the “comprehensive support path”, as it combines multiple conditions that collectively support sustained engagement and self-payment, with the core conditions being the primary drivers of user behavior.

 Configuration 4 represents a simplified path. This configuration illustrates the power of simplicity—users continue to engage because they feel their participation contributes to the societal good, and this intrinsic motivation overrides the need for entertainment or social networking features. This path underscores that users’ sustained participation is driven primarily by the platform’s social value and societal mission, rather than by external entertainment or social influences. In practice, this path would resonate with users who are focused on contributing to social causes, rather than seeking entertainment or social interaction. Platforms adopting this approach can maintain sustained engagement even without heavy reliance on entertainment or social features, as the public value provided becomes the key driver. This path can be aptly named the “pure public value path”, as it emphasizes user engagement based entirely on public value and social mission, rather than entertainment or social networking elements.

#### 5.2.2 Configurations leading to disengagement.

Conversely, the study also identified configurations that are likely to lead to disengagement:

 Configuration 1 underscores the fact that engagement requires a combination of user experience, personal rewards, and social impact—when all these are missing, users will naturally disengage. In practice, this configuration might occur with poorly designed platforms that do not resonate with user expectations, lacking both entertainment and social purpose, resulting in their quick abandonment. This path can be named the “complete disengagement path”, as it represents a scenario where all essential conditions for continued usage are absent.

 Configuration 2 emphasizes the importance of functionality and personal investment in maintaining user engagement. Social factors alone cannot sustain long-term usage, as they are inherently weak without other supporting factors like entertainment or incentives. In the real world, this path might occur in platforms that focus too much on community building without providing the basic conveniences or incentives that users expect. For instance, social apps that lack usability or fun features often struggle to keep users engaged. To mitigate this, platforms must offer functional value and opportunities for personal commitment, such as self-payment mechanisms, to ensure long-term participation. This path is best referred to as the “social impact without incentives path”, reflecting how social influence alone is insufficient without functional support and incentives.

 In configuration 3 highlights the essential role of entertainment in maintaining long-term engagement. Public value and tool convenience alone cannot keep users engaged if the platform fails to offer an enjoyable experience. Without the ability to keep users entertained or offer fulfilling social interactions, platforms will struggle to retain their user base, particularly in a competitive market where entertainment is often a primary draw. The absence of entertainment and social interaction can lead users to feel that the platform is either too utilitarian or lacks the emotional engagement necessary for long-term loyalty. In real-world terms, this path might occur in platforms that are functional but lack the fun, creative elements that make users want to return. This configuration is aptly named the “convenience without enjoyment path”, as it emphasizes the importance of providing a balance between functionality and user enjoyment.

 Configuration 4 underscores the fact that personal investment and social factors cannot sustain long-term engagement without a functional and enjoyable platform. Platforms that focus too heavily on social value and self-payment mechanisms without providing an intuitive or enjoyable experience risk losing their users. This path points out that engagement cannot be maintained on motivation alone—platforms must offer convenience and entertainment alongside social and financial incentives. In the real world, this might happen in platforms where social good is emphasized but usability and fun are neglected. The path can be called the “engagement without enjoyment path”, highlighting the lack of a balanced user experience.

### 5.3 Comparison of SEM and fsQCA

Both SEM and fsQCA highlight the importance of subjective norms in sustaining usage intention, though they conceptualize its influence differently. In SEM, subjective norms serve as an indirect influencer, shaping other factors like self-payment and public value, which then drive engagement. This positions subjective norms as a mediator in the relationship between core factors and sustained usage, with self-payment emerging as a stronger direct predictor of user commitment. This suggests that social pressure and perceived social expectations shape users’ decisions to contribute financially, reinforcing the social dynamics of participation, but they play a less direct role in user retention.

In contrast, fsQCA identifies subjective norms as a core condition for sustained usage. The absence of subjective norms in negation configurations, especially in configurations leading to disengagement, underlines their fundamental role in driving long-term user participation, even when other factors are present. For example, when social influence is lacking, users are less likely to maintain their engagement, even if other factors like tool convenience or public value are present. This highlights the necessity of social validation and social pressure in motivating continued use.

In summary, while SEM provides a precise mapping of factors influencing continuous usage intention, fsQCA adds depth by exploring the interdependencies and conditionality of these factors. The combination of both methods offers a comprehensive understanding of user retention, with SEM highlighting individual drivers and fsQCA revealing the complex configurations that lead to either engagement or disengagement.

## 6 Conclusion

This research integrates the D&M, UTAUT, SDT and SET models in the setting of gamified online public welfare, collectively applying structural equation modeling and fuzzy-set qualitative comparative analysis to uncover factors influencing continuous usage intention.

### 6.1 Theoretical significance

This research posits several contributions to the existing body of theoretical knowledge. Firstly, by adopting continuous usage intention as the perspective, this study integrates the D&M, UTAUT, SDT, and SET models within the context of gamified online public welfare. This fusion not only extends the application scope of these well-established models but also offers a deeper understanding of the multi-dimensional nature of user engagement and retention in the Internet context. The inclusion of SDT and SET provides valuable insights into intrinsic motivation and social exchange dynamics, further enhancing the explanatory power of these models in this specific context. Moreover, the D&M model emphasizes the importance of system quality and information quality in shaping user experience, while UTAUT highlights the significance of performance expectancy, effort expectancy, and social influence in technology adoption, both of which remain relevant in understanding users’ continuous engagement with gamified platforms.

Secondly, the mixed methodology further probes multifaceted interactions to enrich theoretical contributions. Methodologically, our SEM-fsQCA integration establishes a ‘quantitative verification-qualitative interpretation’ paradigm. Future studies should apply SEM to discover dominant relationships, use fsQCA for pattern validation in complex behavioral contexts, and conduct follow-up case studies to operationalize configuration-specific strategies;adopt interpretive complementarity by treating SEM’s β coefficients as indicators of necessary conditions, while interpreting fsQCA’s solution coverage scores as sufficiency thresholds for contextualized implementation; implement practical translation protocols that convert SEM-validated main effects into baseline system requirements, while utilizing fsQCA-derived configurations to develop segment-specific strategies.

Finally, this study also highlights the importance of understanding users’ motivations and behaviors in gamified public welfare activities, which could guide future theoretical and empirical research in this area.

### 6.2 Practical implications

This study offers several actionable recommendations for practitioners designing and managing gamified public welfare platforms. The insights gained from different user configurations can be translated into specific strategies that enhance user engagement, satisfaction, and retention.

First, the positive influence of game design on public value and leisure emphasizes the importance of integrating high-quality game elements that are not only engaging but also promote a sense of social responsibility. Practitioners should focus on creating games with compelling narratives and mechanics that reinforce the social mission of the platform. For example, platforms can design in-game challenges or missions that directly contribute to real-world causes, making it clear to users that their gameplay has a tangible social impact. Furthermore, the significant role of tool convenience in driving public value and self-payment suggests that platforms should prioritize user-friendly interfaces and streamlined interactions. Simplified onboarding processes, intuitive navigation, and easy access to donation features can enhance user satisfaction and make it easier for users to engage in financial contributions. Incorporating social validation features, such as showing users’ contributions to a cause in real time or allowing them to share their achievements with peers, can further encourage self-payment. Additionally, since subjective norms influence self-payment, it would be beneficial for platforms to leverage social influence by integrating peer recognition systems, like leaderboards or badges that highlight users’ charitable actions. By doing so, platforms can create an ecosystem where social pressure, enjoyment, and social responsibility coexist to foster sustained engagement.

In terms of design, the study suggests that platforms should tailor their features and interactions to the specific motivations and behaviors of different user configurations. For platforms aiming to cater to users who value social responsibility over entertainment, the “functionality-driven path” offers a strategy focused on clear societal impact and functional design. These platforms should invest in conveying their social mission effectively through in-game narratives or campaigns that show users how their participation directly contributes to a societal cause. The core design should be simple, functional, and purposeful, with an emphasis on making the social impact easy to understand and act upon. For platforms seeking to attract users who desire both social validation and ease of use, the “comprehensive support path” suggests that a balanced combination of gaming convenience, subjective norms, and self-payment features is essential. In practice, this means ensuring the platform is user-friendly (e.g., providing seamless gameplay and quick donation options), while also integrating mechanisms that foster a sense of social pressure and personal investment. Features like easy payment options, progress tracking, and social sharing are important here. The “pure public value path” highlights the power of intrinsic motivation for societal contribution. Platforms following this path should minimize distractions from entertainment or social interactions and instead focus on communicating the intrinsic value of participation. One strategy could be to highlight real-world benefits, such as how user engagement helps fund a particular cause, ensuring users feel their actions have a direct societal impact. On the other hand, the “complete disengagement path” shows that platforms with no entertainment value or social purpose are likely to lose users quickly. To avoid this, practitioners must ensure the platform offers both functional features and a clear social impact. Similarly, the “social impact without incentives path” indicates that platforms that rely solely on social causes without offering entertainment or rewards will struggle to keep users engaged. Practitioners should therefore integrate features that provide personal incentives, such as gamified elements, achievements, or opportunities for users to see the tangible effects of their contributions. The “convenience without enjoyment path” reveals the need for balance between functional features and user enjoyment. Even though functionality is important, platforms must also ensure the experience is enjoyable, whether through interactive design, aesthetic appeal, or entertainment value. Finally, the “engagement without enjoyment path” highlights the importance of providing both personal investment mechanisms and enjoyable user experiences. Platforms that focus too much on the social good or self-payment features without making the experience fun and easy to use are likely to lose users over time. Therefore, practitioners should prioritize improving user experience by simplifying interfaces, ensuring ease of gameplay, and incorporating enjoyable elements like mini-games, challenges, or social interactions that enhance the overall experience.

### 6.3 Limitations and future research

This study has several limitations that should be considered. First, the cultural context of the sample limits the generalizability of the findings, as the data was primarily collected from a specific geographic area, and cultural differences could affect user engagement with gamified public welfare platforms. Future research could address this by conducting cross-cultural studies to explore how cultural values and social norms influence user behavior across different regions. Second, the sample is disproportionately composed of younger and female participants, which may skew the results. A more balanced and diverse sample would provide a better understanding of how demographic factors such as age, gender, and social background affect engagement. The study also relies on self-reported data, which introduces the possibility of common method bias. To mitigate this, future studies could incorporate multiple data sources, such as behavioral data from platform logs, to validate self-reports. Additionally, the use of cross-sectional data limits the ability to draw causal conclusions, suggesting that future research should consider longitudinal studies to examine how engagement evolves over time. Finally, this study focused on a single platform, limiting its applicability to other contexts. Future research could explore multiple platforms to identify generalizable factors that influence user engagement in game-based charity activities. Addressing these limitations would deepen our understanding of user engagement in gamified public welfare platforms.

## Supporting information

S1 File Questionnaire data. (DOCX)

## References

[1] Wei SJ, Zhang L. Relationship or symbol: An analysis of the factors influencing individual participation in micro public welfare behavior. Journal of Lanzhou University (Social Sciences). 2021;49(3):98–108. doi: 10.13885/j.issn.1000-2804.2021.03.009

[2] Chen KH. Empowering cultural and creative attractions with gamified immersive experiences to drive new changes in the cultural and tourism industry. China Conference & Exhibition. 2021;12:23.

[3] Tedjamulia SJJ, Dean DL, Olsen DR, Albrecht CC. Motivating Content Contributions to Online Communities: Toward a More Comprehensive Theory. In: Proceedings of the 38th Annual Hawaii International Conference on System Sciences. 193b–193b. doi: 10.1109/hicss.2005.444

[4] Tang XL. Research on users’ attention and knowledge contribution quality in social Q&A community. Master’s Thesis, Wuhan University. 2018. Available from: https://kns.cnki.net/kcms2/article/abstract?v=aHgEko1xHjiuJ9f-RzBh0tiAHp2A1qxzFMpQyiSrmxmuawFWqkbHdcddBgygBEFocx1UBGm40jW37Frqp3DPLfX9hSWqHIU5meD0pgnocnjJ524t-ytOmw==&uniplatform=NZKPT&language=gb

[5] Huang JP, Cao AQ. Motivation and incentive mechanism for public participation in knowledge-based crowdsourcing society. Journal of Beijing Institute of Technology (Social Sciences Edition). 2018;20(4):88–96. doi: 10.15918/j.jbitss1009-3370.2018.3253

[6] Zhang LJ. Research on influencing factors of privacy information disclose intention of e-commerce’s users based on cognitive theory of emotion. Master’s Thesis, Central China Normal University. 2023. Available from: https://kns.cnki.net/kcms2/article/abstract?v=aHgEko1xHji0RB-H77c6RmSqV0Jhfwg1Mf81fEvxevyySRI--lXiPXjNwgogHIn3ER12SlMtGJr2GbBZdwCAiJ1blfjPbJMMkfVadcPs7aw08EIZSxvsdg==&uniplatform=NZKPT&language=gb

[7] Song XP, Gong QQ, Yao W. A conceptual model of effective competitive intelligence participation behavior in Web 2.0. Information Studies: Theory & Application. 2017;40(3):5–8. doi: 10.16353/j.cnki.1000-7490.2017.03.002

[8] Mi L, Xu T, Sun Y, Zhao J, Lv T, Gan X, et al. Playing Ant Forest to promote online green behavior: A new perspective on uses and gratifications. J Environ Manage. 2021;278(Pt 2):111544. doi: 10.1016/j.jenvman.2020.111544 33129025

[9] Shao W, Ross M. Testing a conceptual model of Facebook brand page communities. Journal of Research in Interactive Marketing. 2015;9(3):239–58. doi: 10.1108/jrim-05-2014-0027

[10] Chen C-J, Hung S-W. To give or to receive? Factors influencing members’ knowledge sharing and community promotion in professional virtual communities. Information & Management. 2010;47(4):226–36. doi: 10.1016/j.im.2010.03.001

[11] Chiu C-M, Hsu M-H, Wang ETG. Understanding knowledge sharing in virtual communities: An integration of social capital and social cognitive theories. Decision Support Systems. 2006;42(3):1872–88. doi: 10.1016/j.dss.2006.04.001

[12] Hashim KF, Tan FB. The mediating role of trust and commitment on members’ continuous knowledge sharing intention: A commitment-trust theory perspective. International Journal of Information Management. 2015;35(2):145–51. doi: 10.1016/j.ijinfomgt.2014.11.001

[13] Zhang WW, Jiang X. Research on the evolutionary mechanism of continuous participation motivation of online health community users. Chinese Journal of Management. 2020;17(8):1245–53.

[14] Sung Y, Kim Y, Kwon O, Moon J. An Explorative Study of Korean Consumer Participation in Virtual Brand Communities in Social Network Sites. Journal of Global Marketing. 2010;23(5):430–45. doi: 10.1080/08911762.2010.521115

[15] Mao WJ, Liao SS. The impact of member participation in platform governance on user stickiness: A single case study based on BILIBILI. Journal of Management Case Studies. 2020;13(1):71–85.

[16] Lian ZY. Study on the motivation of public participation in the social development project of digital archives resources. Archives Science Study. 2022;4:83–90. doi: 10.16065/j.cnki.issn1002-1620.2022.04.012

[17] Fang CC, Zhang JT. Research of users in social Q&A communities: Commentary and future directions. Journal of Intelligence. 2018;37(9):185–93.

[18] Yuan YX. Research on the game-based mobilization mechanism of Internet public welfare -- Based on the media analysis of “99 Public Welfare Day”. New Media Research. 2022;8(17):115–18. doi: 10.16604/j.cnki.issn2096-0360.2022.17.018

[19] Du SH, Xu JH, Zhang DP, Yang XG. How gamification drives green consumption behavior among e-commerce users: A study on online ethnography based on Ant Forest. Nankai Business Review. 2022;25(2):191–204.

[20] Lee J. Effect of gamification on learners’ class participation motivation and public value. Public Value. 2021;6(4):15–23. doi: 10.22471/value.2021.6.4.15

[21] Xie ZH. A study on user behavior of “Ant Forest” from the perspective of public welfare communication. Research on Transmission Competence. 2019;3(27):210–11.

[22] Zhang Y, Xiao S, Zhou G. User continuance of a green behavior mobile application in China: An empirical study of Ant Forest. Journal of Cleaner Production. 2020;242:118497. doi: 10.1016/j.jclepro.2019.118497

[23] Du HS, Ke X, Wagner C. Inducing individuals to engage in a gamified platform for environmental conservation. IMDS. 2020;120(4):692–713. doi: 10.1108/imds-09-2019-0517

[24] Zhang KX, Wu CQ, Chen XR, Li ML. Integrating education with entertainment: does public participation in environmentally friendly games contribute to regional green development?. China Public Administration Review. 2022;4(3):70–98.

[25] Huang J, Zhou L. Social gamification affordances in the green IT services: perspectives from recognition and social overload. INTR. 2020;31(2):737–61. doi: 10.1108/intr-03-2020-0121

[26] Qin CS, Tian Y. Gamification of environmental protection - can environmentally friendly games change public environmental behavior. China Public Administration Review. 2021;3(2):5–25.

[27] Gao LH, Lu QY. Symbolic capital and symbolic power: A study on the disenchantment of online media’s participation in social welfare. Journalism and Mass Communication. 2017;7:58–62. doi: 10.15897/j.cnki.cn51-1046/g2.2017.07.009

[28] Zhou X. Digital platforms, industry restructuring, and group livelihoods: Taking the changes of vehicle freight matching models in the highway freight market as an example. Sociological Studies. 2021;36(5):47–69 + 227.

[29] Wu CQ, Shi L, Zhang KX. Research on the internationalization of crowdfunding platforms under the background of digital economy: From the perspective of value co-creation. Research on Economics and Management. 2020;41(6):3–18. doi: 10.13502/j.cnki.issn1000-7636.2020.06.001

[30] Liu X, Chen S, Xiong L. Research on user donation preference of “Internet + Public Benefit” project – Take Taobao Public Welfare as an example. Int J Soc Sci Educ Res. 2020;3(1):72–8.

[31] Guo GQ, Liu RJ, Wang JG. Research on the inner mechanism of Ant Forest users turning to offline green consumption: Based on the perspective of behavioral reasoning theory. Journal of Management. 2023;36(1):56–69. doi: 10.19808/j.cnki.41-1408/F.2023.0005

[32] DeLone WH, McLean ER. The DeLone and McLean model of information systems success: A ten-year update. Journal of Management Information Systems. 2003;19(4):9–30. doi: 10.1080/07421222.2003.11045748

[33] Yang GF. A study on the factors influencing user satisfaction and continuous use intention in mobile reading – using content aggregation apps as an example. Journal of Modern Information. 2015;35(3):57–63.

[34] He DC, Deng B, Liu QC. Research on continuous use intention of community group buying users based on ECM and D&M models. Times of Economy & Trade. 2023;20 (1):103–7. doi: 10.19463/j.cnki.sdjm.2023.01.029

[35] Yang YW, Sun GH, Wang Y. Are consumers willing to adopt recommendations—— based on the information system success technology acceptance model. Journal of Central University of Finance & Economics. 2016;7:109–17.

[36] Venkatesh M, Davis D. User Acceptance of Information Technology: Toward a Unified View. MIS Quarterly. 2003;27(3):425. doi: 10.2307/30036540

[37] Ming JR, Zhang J, Yang YN, Chen KL. A user behavior model and empirical study of mobile library based on UTAUT. Library Tribune. 2017;37(6):70–7.

[38] Hu DH, Zhang YF. Study on the influencing factors of health app usage among college students based on UTAUT. Library. 2019;3:63–8.

[39] Ma XL, Liu LJ. Research on the influencing factors of urban residents’ willingness to use Internet social wealth management products based on UTAUT. Consumer Economics. 2016;32(2):81–5.

[40] Liu JD, Li QX, Wang J. Research on factors influencing user behavior of online office app based on UTAUT model. Information Science. 2020;38(9):49–55 + 68. doi: 10.13833/j.issn.1007-7634.2020.09.008

[41] Zhang K, Zhang P, Zhang Y. A study on the influencing factors and behaviors of tourism app user use based on UTAUT and TTF theory. Enterprise Economy. 2016;9:150–6. doi: 10.13529/j.cnki.enterprise.economy.2016.09.024

[42] Zhou T, Lu YB, Zhang JL. Research on user adoption behavior of mobile banking from the perspective of integrating TTF and UTAUT. Journal of Management Science. 2009;22(3):75–82.

[43] Deci EL, Ryan RM. Self-determination theory. In: Van Lange PA, Kruglanski AW, Higgins ET, Editors. Handbook of theories of social psychology. Thousand Oaks (CA): Sage Publications. 2012. p. 416–36.

[44] Chiu TKF, Sun JCY, Ismailov M. Investigating the relationship of technology learning support to digital literacy from the perspective of self-determination theory. Educ Psychol. 2022;42(10):1263–82.

[45] Ryan RM, Rigby CS, Przybylski A. The motivational pull of video games: A self-determination theory approach. Motiv Emot. 2006;30:344–60.

[46] Sheldon KM, Titova L. Social media use and well-being: testing an integrated self-determination theory model. Media Psychology. 2023;26(6):637–59. doi: 10.1080/15213269.2023.2185259

[47] Jeno LM, Egelandsdal K, Grytnes J-A. A qualitative investigation of psychological need-satisfying experiences of a mobile learning application: A Self-Determination Theory approach. Computers and Education Open. 2022;3:100108. doi: 10.1016/j.caeo.2022.100108

[48] Homans GC. Social behavior as exchange. Am J Sociol. 1958;63(6):597–606.

[49] Nasrolahi Vosta L, Jalilvand MR. Electronic trust-building for hotel websites: a social exchange theory perspective. J Islam Mark. 2023;14(11):2689–714.

[50] Li J. Knowledge sharing in virtual communities: A social exchange theory perspective. J Ind Eng Manag. 2015;8(1):170–83.

[51] Urbonavicius S, Degutis M, Zimaitis I, Kaduskeviciute V, Skare V. From social networking to willingness to disclose personal data when shopping online: modeling in the context of social exchange theory. J Bus Res. 2021;136:76–85.

[52] Chou S-W, Hsu C-S. Understanding online repurchase intention: social exchange theory and shopping habit. Inf Syst E-Bus Manage. 2015;14(1):19–45. doi: 10.1007/s10257-015-0272-9

[53] Boomsma A. The robustness of maximum likelihood estimation in structural equation models. Structural Modeling by Example. Cambridge University Press. 1988. p. 160–88. doi: 10.1017/cbo9780511601118.010

[54] Zha JX, Wang LS. Empirical study of influential elements of e-satisfaction. Journal of Management Science. 2006;1:50–8.

[55] Chen TX, Yao M. Factors influencing individual donations to nonprofit organizations: A questionnaire survey in Guangzhou. Journal of Zhejiang University (Humanities and Social Sciences). 2012;42(4):114–31.

[56] Zhang X, Zhao Y, Xiao Q. A study on the factors influencing individual donation intention in social network context. Journal of Information and Management. 2019;4(1):19–29.

[57] Poels K, de Kort YD, IJsselsteijn WI. D3.3: Game Experience Questionnaire. Brussels: European Commission. 2007.

[58] Chen XR, Yang L, Zhang RL, Zhang KX. How gamification operations of technology platforms drive public welfare development: An analysis perspective based on virtual donation. Youth Studies. 2023;1:58–68.

[59] Deterding S, Dixon D, Khaled R, Nacke L. From game design elements to gamefulness. In: Proceedings of the 15th International Academic MindTrek Conference: Envisioning Future Media Environments, 2011. 9–15. doi: 10.1145/2181037.2181040

[60] Santos FC, Santos MD, Pacheco JM. Social diversity promotes the emergence of cooperation in public goods games. Nature. 2008;454(7201):213–6. doi: 10.1038/nature06940 18615084

[61] Johnson D, Wiles J. Effective affective user interface design in games. Ergonomics. 2003;46(13–14):1332–45. doi: 10.1080/00140130310001610865 14612323

[62] Vives X. Endogenous public information and welfare in market games. Rev Econ Stud. 2017;84(2):935–63.

[63] Boontarig W, Chutimaskul W, Chongsuphajaisiddhi V, Papasratorn B. Factors influencing the Thai elderly intention to use smartphone for e-Health services. In: 2012 IEEE Symposium on Humanities, Science and Engineering Research, 2012. 479–83. Available from: doi: 10.1109/shuser.2012.6268881

[64] Zhang CL, Wang XW, Wang CX. A study on the factors influencing the continuous information sharing behavior of network community users. Information and Documentation Services. 2019;40(3):53–62.

[65] Huang F, Wang J. A consumer acceptance behavior model based on perceived value and empirical research. Commercial Research. 2013;6:19–27. doi: 10.13902/j.cnki.syyj.2013.06.001

[66] Skelly TAM, Jensen KH, Halkier B. The convenience of gaming. Consumption and Society. 2023;2(1):102–21.

[67] Yu L, Chen Z, Yao P, Liu H. A study on the factors influencing users’ online knowledge paying-behavior based on the UTAUT model. Journal of Theoretical and Applied Electronic Commerce Research. 2021;16(5):1768–90.

[68] Lu C-C, Tsai-Lin T-F. Are Older Adults Special in Adopting Public eHealth Service Initiatives? The Modified Model of UTAUT. Sage Open. 2024;14(1). doi: 10.1177/21582440241228639

[69] Sun Y, Wang N, Guo X, Peng Z. Understanding the acceptance of mobile health services: A comparison and integration of alternative models. J Electron Commer Res. 2013;14:183.

[70] Saprykin D, Kurcheeva G, Bakaev M. Impact of social media promotion in the information age. In: Proceedings of the International Conference on Electronic Governance and Open Society: Challenges in Eurasia, 2016.

[71] Zhou T, Li H. Understanding mobile SNS continuance usage in China from the perspectives of social influence and privacy concern. Computers in Human Behavior. 2014;37:283–9. doi: 10.1016/j.chb.2014.05.008

[72] Ampt AJ, Amoroso C, Harris MF, McKenzie SH, Rose VK, Taggart JR. Attitudes, norms and controls influencing lifestyle risk factor management in general practice. BMC Fam Pract. 2009;10:59. doi: 10.1186/1471-2296-10-59 19706198 PMC2746183

[73] Cialdini RB, Goldstein NJ. Social influence: compliance and conformity. Annu Rev Psychol. 2004;55:591–621. doi: 10.1146/annurev.psych.55.090902.142015 14744228

[74] Lin QW, Zhu XL. A study on the motivation of young people to participate in the “Ant Forest” public welfare game: Based on the octagonal behavior analysis model. Youth and Adolescent Studies. 2022;5:49–55. doi: 10.16399/j.cnki.qsnyj.2022.05.002

[75] Pan PQ. Research on user interaction behavior and motivation of Ant Forest. New Media Research. 2023;9(1):51–6. doi: 10.16604/j.cnki.issn2096-0360.2023.01.024

[76] Ping Ting M, Dong Min C. What drives user churn in serious games? An empirical examination of the TAM, SOR theory, and game quality in Chinese cultural heritage games. Entertainment Computing. 2025;52:100758. doi: 10.1016/j.entcom.2024.100758

[77] Korn O, Schmidt A. Gamification of Business Processes: Re-designing Work in Production and Service Industry. Procedia Manufacturing. 2015;3:3424–31. doi: 10.1016/j.promfg.2015.07.616

[78] Bhagat S, Jeong EJ, Kim DJ. The Role of Individuals’ Need for Online Social Interactions and Interpersonal Incompetence in Digital Game Addiction. International Journal of Human–Computer Interaction. 2019;36(5):449–63. doi: 10.1080/10447318.2019.1654696

[79] Davis FD. Perceived Usefulness, Perceived Ease of Use, and User Acceptance of Information Technology. MIS Quarterly. 1989;13(3):319. doi: 10.2307/249008

[80] AbuShanab E, Pearson JM. Internet banking in Jordan: The unified theory of acceptance and use of technology (UTAUT) perspective. Journal of Systems and Information Technology. 2007;9(1):78–97.

[81] Turel O, Serenko A, Giles P. Integrating technology addiction and use: An empirical investigation of online auction users. MIS Quarterly. 2011;35(4):1043–61.

[82] Hair JF, Risher JJ, Sarstedt M, Ringle CM. When to use and how to report the results of PLS-SEM. European Business Review. 2019;31(1):2–24.

[83] Fornell C, Larcker DF. Evaluating structural equation models with unobservable variables and measurement error. Journal of Marketing Research. 1981;18(1):39–50.

[84] Hair Jr JF, Hult GTM, Ringle CM, Sarstedt M, Danks NP, Ray S. Partial least squares structural equation modeling (PLS-SEM) using R: A workbook. Springer Nature. 2021.

[85] Schneider CQ, Wagemann C. Set-Theoretic Methods for the Social Sciences. Cambridge University Press. 2012. doi: 10.1017/cbo9781139004244

[86] Fiss PC. Building Better Causal Theories: A Fuzzy Set Approach to Typologies in Organization Research. AMJ. 2011;54(2):393–420. doi: 10.5465/amj.2011.60263120

[87] Greckhamer T, Furnari S, Fiss PC, Aguilera RV. Studying configurations with qualitative comparative analysis: Best practices in strategy and organization research. Strategic Organization. 2018;16(4):482–95. doi: 10.1177/1476127018786487

[88] Wang X, Yao X. Fueling pro-environmental behaviors with gamification design: identifying key elements in ant forest with the kano model. Sustainability. 2020;12(6):2213.

[89] Wertz RJ. Reality is broken—why games make us better and how they can change the world. Journal of Communications Media Studies. 2011;3(1):174–6.

[90] Rapp A. Social Game Elements in World of Warcraft: Interpersonal Relations, Groups, and Organizations for Gamification Design. International Journal of Human–Computer Interaction. 2018;34(8):759–73. doi: 10.1080/10447318.2018.1461760

[91] Douglass RB. Belief, attitude, intention, and behavior: An introduction to theory and research. 1977.

[92] Venkatesh V, Thong JYL, Xu X. Consumer Acceptance and Use of Information Technology: Extending the Unified Theory of Acceptance and Use of Technology. MIS Quarterly. 2012;36(1):157. doi: 10.2307/41410412

[93] Hamari J, Koivisto J, Sarsa H. Does gamification work?—A literature review of empirical studies on gamification. In: Proceedings of the 2014 47th Hawaii International Conference on System Sciences. IEEE; 2014. p. 3025–34.

[94] Cecere G, Le Guel F, Rochelandet F. Crowdfunding and social influence: an empirical investigation. Applied Economics. 2017;49(57):5802–13. doi: 10.1080/00036846.2017.1343450

[95] Hofstede G. Culture’s consequences: Comparing values, behaviors, institutions and organizations across nations. Thousand Oaks: Sage Publications. 2001.

[96] Jackson LA, Wang J-L. Cultural differences in social networking site use: A comparative study of China and the United States. Computers in Human Behavior. 2013;29(3):910–21. doi: 10.1016/j.chb.2012.11.024

[97] Lin Z, Xu H. Estimation of social-influence-dependent peer pressure in a large network game. The Econometrics Journal. 2017;20(3):S86–102. doi: 10.1111/ectj.12102

[98] Deci EL, Ryan RM. The “What” and “Why” of Goal Pursuits: Human Needs and the Self-Determination of Behavior. Psychological Inquiry. 2000;11(4):227–68. doi: 10.1207/s15327965pli1104_01

