# Supplementary material for: How to influence the continuous usage intention of game-based Internet public welfare users? An empirical analysis based on SEM and fsQCA
Source: PLoS One. 2025 Jun 11;20(6):e0325933. doi: 10.1371/journal.pone.0325933 (PMC12157838; doi:10.1371/journal.pone.0325933)
Supplement: S1 File — (DOCX) [file pone.0325933.s001.docx]

**Appendix:** **Survey Questionnaire**

*Hello! Thank you for participating in this survey, which aims to better understand the factors the influence the usage intention of game-based Internet public welfare users. Please take a few minutes to complete the following questions. Your honest responses will help inform our research and contribute to the development of relevant initiatives.*

*To indicate your answer please* **CIRCLE** *the number alongside the answer that comes closest to your opinion or write your answer in the space provided.*

*The survey data will only be used for academic research without any commercial purpose. Your answers will be kept strictly confidential.The entire survey process fully complies with ethical requirements. If you are unwilling to participate in the survey, you can exit the system. If you agree to participate in the survey, please proceed to the next step.Thank you for your support and participation!*

**Please indicate your information.**

Your gender:1.Male; 2.Female

Your age group: 1.Under 18; 2.18 to 25; 3.26 to 30; 4.31 to 40; 5.41 to 50; 6.51 to 60; 7.Over 60

Your current occupation: 1.Full-time student; 2.Office worker; 3.Freelancer; 4.Homemaker; 5Retired

How long have you been using charity games:1.Within 1 year; 2.1-2 years; 3.More than 3 years; 4.Do not useName(optional):

*The following questions are based on the Likert 5-level scale. Please choose your level of agreement based on the viewpoint expressed in the question: 1. Completely disagree; 2. Disagree; 3. Neutrality; 4. Identification; 5. Fully agree.* ***Please try to avoid selecting the "neutral" option as much as possible.***

| **Q1: Game Design** | | | | | |
| --- | --- | --- | --- | --- | --- |
| Public welfare game function design and page layout is reasonable and beautiful. | 1 | 2 | 3 | 4 | 5 |
| Public welfare games are simple and intuitive to operate. | 1 | 2 | 3 | 4 | 5 |
| Public welfare games can always show the whole process of public welfare. | 1 | 2 | 3 | 4 | 5 |
| **Q2: Tool Convenience** | | | | | |
| I have smart devices for charity games. | 1 | 2 | 3 | 4 | 5 |
| I can play charity games anytime and anywhere. | 1 | 2 | 3 | 4 | 5 |
| The smart device I use is compatible with charity games. | 1 | 2 | 3 | 4 | 5 |
| **Q3: Gaming Convenience** | | | | | |
| I prefer to choose well-known public service games. | 1 | 2 | 3 | 4 | 5 |
| I can find help if I have a problem. | 1 | 2 | 3 | 4 | 5 |
| Charity games are rich and diverse in content. | 1 | 2 | 3 | 4 | 5 |
| **Q4: Social Factors** | | | | | |
| I use charity games through my friends. | 1 | 2 | 3 | 4 | 5 |
| I use charity games to promote them through media advertising. | 1 | 2 | 3 | 4 | 5 |
| I use charity games because they come with major software. | 1 | 2 | 3 | 4 | 5 |
| **Q5: Subjective Norms** | | | | | |
| I think the charity games are very interesting. | 1 | 2 | 3 | 4 | 5 |
| I have a sense of responsibility for the public good. | 1 | 2 | 3 | 4 | 5 |
| Charity games can give me a sense of accomplishment. | 1 | 2 | 3 | 4 | 5 |
| **Q6: Public Value** | | | | | |
| I can contribute to the public good. | 1 | 2 | 3 | 4 | 5 |
| I can increase my public welfare contribution (environmental protection certificate, donation value, etc.) via charity games. | 1 | 2 | 3 | 4 | 5 |
| Charity games can stimulate my enthusiasm for public welfare. | 1 | 2 | 3 | 4 | 5 |
| **Q7: Leisure and Entertainment** | | | | | |
| I can relax and unwind through charity games. | 1 | 2 | 3 | 4 | 5 |
| I can interact with my friends through charity games. | 1 | 2 | 3 | 4 | 5 |
| I can enrich my daily life through public welfare games. | 1 | 2 | 3 | 4 | 5 |
| **Q8: Self-Payment** | | | | | |
| I can quickly adapt to charity games. | 1 | 2 | 3 | 4 | 5 |
| I can use my fragmented time to participate in charity games. | 1 | 2 | 3 | 4 | 5 |
| I can explore other useful activities in real life through charity games. | 1 | 2 | 3 | 4 | 5 |
| **Q9: Continuous Usage Intention** | | | | | |
| In the future, I plan to continue to use charity games. | 1 | 2 | 3 | 4 | 5 |
| In the future, I will maintain or even increase the frequency with which I use charity games. | 1 | 2 | 3 | 4 | 5 |
| I would like to recommend charity games to the people around me. | 1 | 2 | 3 | 4 | 5 |

*Thank you for completing the questionnaire! Your feedback is valuable to us. Have a great day!*

**Note:** This is a paper version of the survey questionnaire for this study. We have published an electronic version of the questionnaire with the same content on the Questionnaire Star platform (www.wjx.cn), but the style may be different.
